# Prevalence and determinants of recent HIV testing among older persons in rural Uganda: a cross-sectional study

**DOI:** 10.1186/s12889-020-8193-z

**Published:** 2020-01-31

**Authors:** Stephen Ojiambo Wandera, Betty Kwagala, Fred Maniragaba

**Affiliations:** 1 Department of Population Studies, School of Statistics and Planning, College of Business and Management Sciences, Makerere University, Kampala, Uganda; 2 Demography and Population Studies Programme, Schools of Social Sciences and Public Health, University of the Witwatersrand, Witwatersrand, South Africa

**Keywords:** HIV, AIDS, HIV testing, Aging, STIs, Uganda, Africa

## Abstract

**Background:**

There is limited research on HIV testing among older persons in Uganda. The aim of this study was to investigate the socio-demographic determinants of recent HIV testing among older persons in selected rural districts in Uganda.

**Methods:**

A cross-sectional survey of 649 older men and women age 50 years and older, from central (Masaka district) and western (Hoima district) Uganda was conducted. Frequency distributions, chi-square tests and multivariable logistic regressions were used to examine the association between recent HIV testing and selected explanatory variables.

**Results:**

Nearly six in ten (58%) of older persons had primary education. About 60% of the respondents were in union and 13% of them had two or more spouses. Half of the older people (51%) had sex in the last twelve months. A quarter (25%) of older persons gave or received gifts in exchange for sex in their lifetime. Nearly a third (29%) reported sexually transmitted infections in the last 12 months. Prevalence of lifetime HIV testing was 82% and recent (last 12 months) HIV testing was 53%. HIV testing in the last 12 months was associated with age (OR = 0.50; 95% CI: 0.31–0.79), self-reported sexually transmitted infections (OR = 1.59; 95% CI: 1.00–2.30), male circumcision (OR = 1.71; 95% CI: 1.0–2.93), and sexual activity in the last 12 months (OR = 2.89; 95% CI: 1.83–4.57).

**Conclusion:**

Recent HIV testing among older persons was associated with younger age, self-reported STIs, male circumcision, and sexual activity among older persons in rural Uganda. HIV testing interventions need to target older persons who are 70 years and older, who were less likely to test.

## Background

The African Union framework on Ageing (AU-Plan) defines older persons as those age 60 years and older [1]. In Uganda, during the drafting of the policy for older persons in 2009, age 60 years and older was used [2, 3]. In this study, we used the World Health Organization (WHO) recommendation of using age 50 and older to define older persons [4]. Subsequently, several studies adopted age 50 and older, as an appropriate definition of old age in sub-Saharan Africa (SSA) countries including Kenya [5, 6]; Uganda [7] and South Africa [8] and those of the WHO Study on global AGEing and adult health (SAGE) and the INDEPTH network [9–14].

Globally, the proportion of older persons (age 60 years and older) in 2017 was 13% of the total population [15]. This proportion is projected to increase to 30% by 2050 [16]. Globally, the absolute number of older people increased from 205 million in 1950 to 810 million in 2012 and is anticipated to increase further to 1 billion by 2022 and to 2 billion by 2050 – outnumbering children aged 0–14 years [17–19]. The highest proportion of older persons is projected to live in developing countries by 2050 [20].

In Uganda, the proportion of older persons age 50 years and older was estimated at 7.6% in the 2014 Uganda Population and Housing Census (UBOS, 2014). There has been some growth in the absolute number of older people in Uganda from 1.1 million in 2002 (4.5% of the population) to 1.3 million in 2010 (out of 30 million) and is expected to increase to 5.5 million (constituting 5.7% of the population) by 2050 [3, 18].

HIV in old age is an emerging public health challenge [21] and considered as a “hidden epidemic” globally [22]. In SSA, only 45% of the general population who are HIV positive know their status [23, 24]. This is far from the UNAIDS ambitious target of ensuring that 90% of those who are HIV positive know their status [25]. With regard to older persons, it is not easy to estimate the prevalence of HIV and the proportion of those who know their HIV status in SSA in general and Uganda in particular [26]. HIV in old age has two major pathways namely; ageing with HIV and infections in old age (Scholten et al., 2011). Ageing with HIV is attributed to adherence to antiretroviral therapy (ART) [27–29].

Prevalence of HIV among older persons is estimated at 11–13% globally [22, 30]. In sub-Saharan Africa, ten countries (mostly in southern and eastern Africa) account for 80% of all people living with HIV. These include: South Africa (25%), Nigeria (13%), Mozambique (6%), Uganda (6%), Tanzania (6%), Zambia (4%), Zimbabwe (6%), Kenya (6%), Malawi (4%) and Ethiopia (3%) [31–34].

HIV testing varies across counties. Among older persons, HIV testing was nearly half (48%) in Uganda [26], 54% in South Africa [35], and 23% in Zimbabwe [36]. However, HIV testing programmes do not prioritize older persons. Yet many lack information on HIV prevention and rarely test for HIV/AIDS [31, 37, 38]. In addition, older persons experience stigma while accessing HIV testing services [37].

The determinants of HIV testing among older persons have been summarized in the conceptual model adapted from the healthcare utilization model. These include predisposing factors (age, gender, race / ethnicity, education, household income, employment status), enabling factors (health insurance, access to care, previous testing, seeing a doctor) and need factors especially HIV risk behaviours in the past 12 months [39–42].

Gender is a significant determinant of HIV testing among older persons, where older women are more likely to test for HIV compared to older men [43]. Among older persons, advanced age has been associated with reduced odds of HIV testing [44]. The perceived risk of contracting HIV is an important predictor of HIV testing, where a low perceived risk is associated with reduced odds of HIV testing [45]. Among older persons, prior history of testing reduces the odds of subsequent HIV testing [44].

A high level of education increases the odds of HIV testing among older people. With regard to marital status, HIV testing is higher among ever married persons outside union i.e. divorced or separated or widowed [26]. In addition, fear, emotional stress of positive HIV sero status results, HIV stigma [46] and knowledge of HIV transmission [37, 47] have been associated with HIV testing [37].

Despite the available evidence on HIV testing, there is dearth of information on HIV testing among older persons in developing countries in general and Uganda [48] in particular. In SSA, sources of data on HIV/AIDS such as Demographic and Health Surveys (DHS) and AIDS Indicator Surveys focus on age 15–54 years. The recent Uganda DHS, 2016 and Population and HIV Impact Surveys (PHIA) in 12 African countries also omit older persons [29, 33, 49]. In addition, several studies on the health of older people in Uganda have focused on later life problems associated with HIV/AIDS (Scholten et al., 2011; Seeley, Wolff, Kabunga, Tumwekwase, & Grosskurth, 2009) but not their uptake of HIV testing. There is need for language editing.

Therefore, the aim of this study was to investigate the determinants of access to HIV testing services among older persons in selected rural districts in Uganda. Findings are expected to contribute to understanding of factors associated with HIV testing among older persons [37, 38, 44, 50].

## Methods

### Study design and setting

The study used a cross-sectional and mixed methods study design. Both survey data and qualitative data were collected in February 2018. Qualitative data included focus group discussions and in-depth interviews. However, this paper is based on survey results. The study setting was in rural districts of Uganda: Masaka (central Uganda) and Hoima (western Uganda).

### Study participants

Older persons were defined as those age 50 years and older as recommended by the World Health Organization [4]. We thus considered older persons 50 years and above who had the capacity to provide informed consent.

### Sampling procedures

We used a multi-stage stratified cluster sampling design. We randomly selected two regions in Uganda namely: central and western regions out of the four administrative ones. Simple random sampling was used to select one district from each region: Masaka (central) and Hoima (western) from each region. We used the sampling frame of the 2014 Uganda Population and Housing Census [51] to select two sub-counties from Masaka [52] and three sub-counties from Hoima [53] using simple random sampling. From each sub-county, four enumeration areas or villages were selected using simple random sampling. The census sampling frame listed all enumeration areas in each sub-county. Using Microsoft office excel package, computer generated random numbers were assigned to all enumeration areas. Random numbers were used to randomly select enumeration areas. From each village, a sampling frame of older persons’ and their households was constructed in consultation with local leaders and systematic sampling was used to select participants for the survey. In households where older men and women live as couples, both were interviewed separately.

Kish’s formula [54] was applied to generate a sample size of 649 older persons for the survey. The prevalence of HIV testing for those age 50–59 years was 45% among men and 49% among women [55]. We used the lower bound of HIV testing (45%), the *p* = 0.45 and the q = 0.55. The level of confidence was set at 95% (z = 1.96) and the error at 8% (e = 0.008). The expected sample size was 148.5. The sample size was multiplied by the design effect of two (D = 2). Therefore, the expected sample size was 297. The final sample size after adjusting for a response rate of 90% became 330. To allow for small area (district) estimations, the sample size was multiplied by two since the study covered two districts. The overall sample size was 660 older persons. Due to non-response, the final sample size was 649 older persons. The number of older persons selected from each enumeration area was determined by probability proportionate sampling (PPS) from the 2014 Uganda census sampling frame [51].

### Data collection procedures

Research assistants were trained for three days by the principal investigator. A pilot study / pre-testing of the tools was conducted in Wakiso town council. Feedback from pilot was integrated in revision of the tools – survey questionnaire and guides for qualitative data. The survey questionnaire was programmed in Android enabled tablets and the SurveyCTO online platform. The survey was conducted in Hoima and Masaka in February 2018. Two research teams (composed of five research assistants) were sent to each district at the same time to collect survey and qualitative data.

### Data management using SurveyCTO

Survey data were collected using SurveyCTO [56] application installed on android enabled Tablets. Data were downloaded from the SurveyCTO Server as STATA files on daily basis.

### Outcome variable

The outcome variable was recent HIV testing (last 12 months). Participants were asked if they had ever an HIV test in their life time (yes = 1, no =0) and if they tested for HIV in the last 12 months (yes =1 or no = 0). A follow up question was about reception of HIV results during the recent HIV test (yes or no responses). A binary variable called recent HIV testing was recoded to a binary variable (yes and no). Recent HIV testing means HIV testing and reception of results in the last 12 months.

### Explanatory variables

Demographic variables included age, and sex. Age was recoded into three categories: 50–59, 60–69 and 70 and older. Sex was recoded into male and female.

Socio-economic variables included: education level (none, primary and secondary or higher), working in the last 12 months (yes and no), religion, marital status or currently in union, number of other wives, and children ever born and those currently alive. Religion was recoded as Catholics, Anglicans, Muslims and Others.

HIV related variables included knowledge about HIV transmission, HIV stigma, and need to test for HIV (yes or no). These questions were adopted from the Population HIV Impact Assessment (PHIA) survey [33] and the Demographic and Health survey [55]. To measure correct knowledge about HIV transmission, five questions were asked:

1. Can the risk of HIV transmission be reduced by having sex with only one uninfected partner who has no other partners? (coded as yes =1, no = 0).
2. A person can get HIV from mosquito bites? (coded as yes =0, no = 1).
3. Can a person reduce their risk of getting HIV by using a condom every time they have sex? (coded as yes =1, no = 0).
4. A person cannot get HIV by sharing food with someone who has HIV? (coded as yes =1, no = 0).
5. Can a healthy-looking person have HIV? (coded as yes =1, no = 0).

These questions were recoded as binary variables. Then they were added together to generate an aggregate variable for correct HIV knowledge. The five statements of HIV knowledge had a low reliability test (Cronbach’s alpha of 0.43). Correct knowledge about HIV transmission was categorized as agreement to at least four to five statements. Those who had agreement to none to three statements were recoded as not having correct knowledge on HIV transmission.

HIV stigma was measured by eight binary (coded as yes = 1 and no = 0) statements (Cronbach’s alpha was 0.60):

1. Would not buy fresh vegetables from an HIV positive vendor
2. Children living with HIV should not be allowed to attend school with children who do not have HIV
3. People hesitate to take an HIV test because they are afraid of how other people will react if the test result is positive
4. People talk badly about people living with HIV, or who are thought to be living with HIV
5. People living with HIV, or thought to be living with HIV, lose the respect of other people
6. Fear that one could get HIV if in contact with the saliva of a person living with HIV
7. Would be ashamed if someone in family had HIV
8. Not willing to care for someone living with HIV

These HIV stigma statements were recoded into binary form and added together to form a score (range from 0 to 8). A binary variable called stigma on at least four statements was created (0 = agreement on 0–3 statements; 1 = agreement to 4–8 statements).

HIV related behaviour included sexual activity in the last 12 months (yes or no), number of life time sexual partners, transactional sex (life time and recent), alcohol consumption, male circumcision and self-reported STIs. Transactional sex involved giving and receiving of gifts for sex in the last 12 months. Substance use variables included alcohol consumption, smoking and use of tobacco. Males were asked to report about their circumcision status (yes, no and not applicable for females).

Self-reported STIs were measured by asking four questions:

1. During the last 12 months, have you had an abnormal discharge from your vagina or experienced pelvic pain (if woman) or penis (if man)?
2. During the last 12 months, have you had an ulcer or sore on or near your vagina (woman) or penis (man)?
3. During the last 12 months, have you had pain on urination?
4. In the last 12 months, did a doctor, clinical officer or nurse tell you that you had a sexually transmitted disease other than HIV?

These questions had three categories (yes, no and don’t know). The Cronbach’s alpha for the four statements was 0.71. The “don’t know” category was merged with the “No” category in order to create binary variables to allow creation of an aggregate variable. In addition, the responses to the former were few. After, an aggregate variable – self-reported STIs was created for those who reported an abnormal discharge, ulcer or sore in the genital area, pain during urination and were told to have an STI by a health provider.

### Statistical analysis

Frequency distributions were used to describe the background characteristics of the older persons. Cross-tabulations were used to investigate associations between recent HIV testing (outcome variable) and selected explanatory variables. Pearson’s chi-squared (χ^2^) tests were used to examine the significant differences between recent HIV testing and the explanatory variables. The level of statistical significance using *p*-values was set at *p* < 0.05.

Multivariable logistic regression analyses were used to examine the association between recent HIV testing and explanatory variables whose p-values were less than 0.05 during the chi-square tests. We used a step-wise regression for multivariable analysis. The first model includes respondents’ background characteristics. In the second model, we added HIV knowledge and attitude factors, and in the third and final model we added behavior factors. Results are presented in the form of Odds Ratios (OR) reporting 95% confidence intervals. The level of statistical significance using *p*-values was set at *p* < 0.05. All analyses were performed in STATA version 15.

## Results

### Descriptive characteristics

Table 1 shows the descriptive characteristics of older persons in rural Uganda. About 52% of the respondents were female and 52% were 60 years and older. The majority (75%) had primary or no formal education, were working (53%), either Catholic or Anglican (75%), and were either married or cohabiting (60%).

**Table 1:** Descriptive characteristics of older persons in rural Uganda

Variables	Number (n)	Percent (%)
Age group		
50–59	312	48.3
60–69	176	27.2
70+	158	24.5
Sex of the respondents		
Female	334	51.5
Male	315	48.5
Education level		
None	111	17.1
Primary	378	58.2
Secondary or higher	160	24.7
Worked in the last 12 months		
No	308	47.5
Yes	341	52.5
Religion		
Catholic	279	43.0
Anglican	208	32.0
Muslim	99	15.3
Others	63	9.7
Currently in union		
No	262	40.4
Yes	387	59.6
Number of wives or husbands		
One	304	46.8
Two plus	83	12.8
Not in union	262	40.4
Correct knowledge about 4–5 HIV transmission statements		
No	230	35.4
Yes	419	64.6
Stigma on at least 4–8 statements		
No	436	67.2
Yes	213	32.8
Total	649	100.0
Need to test for HIV once a year even when you know you are HIV negative		
No	35	5.4
Yes	614	94.6
Do older men who are 50 years older need to be circumcised to prevent HIV in		
No	202	31.1
Yes	447	68.9
Had sex in the last 12 months		
No	314	49.5
Yes	320	50.5
Number of lifetime sexual partners		
One	231	72.4
Two or more	88	27.6
Ever given or received money or gifts for sex		
No	490	75.5
Yes	159	24.5
Gave or received money or gifts for sex in the last 12 months		
No	574	88.4
Yes	75	11.6
Drinks alcohol		
No	426	65.6
Yes	223	34.4
Uses tobacco or drugs		
No	564	86.9
Yes	85	13.1
Self-reported STI in the last 12 months		
No	459	70.7
Yes	190	29.3
Male circumcised		
No	199	30.7
Yes	116	17.9
No, female	334	51.5
Ever tested for HIV and received results		
No	119	18.3
Yes	530	81.7
Tested for HIV and received results in the last 12 months		
No	307	47.3
Yes	342	52.7
Total	**649**	**100**

With respect to HIV knowledge and attitude factors, the majority (65%) had correct knowledge on 4–5 HIV transmission statements, had less or no HIV associated stigma (67%), agreed to the need to test for HIV once a year (95%), and the need for male circumcision to prevent HIV (69%). About half (51%) had sex in the past 12 months. Majority (72%) had had one lifetime sexual partner. On the other hand, a small proportion of older people had received or given money or gifts for sex (24%) in their lifetime and in the last 12 months preceding the study (13%), drunk alcohol (34%) and used tobacco or drugs (13%). Some of the respondents reported suffering from STIs in the past 12 months (29%) and were circumcised (17%). Slightly over half (53%) and over three quarters (82%) tested for HIV in the last 12 months and in their lifetime, respectively.

### Association between HIV testing in the past 12 months and independent factors

Table 2 shows the association between recent HIV testing and reception of results and background factors among older persons in Uganda.

**Table 2:** Association between recent HIV testing and background factors among older persons in Uganda

Variables	Tested for HIV and received results in the last 12 months		
	Yes (%)	Total (N)	*P-value*
Age group			**< 0.001**
50–59	63.5	312	
60–69	50.6	176	
70+	34.2	158	
Sex of the respondents			0.21
Female	50.3	334	
Male	55.2	315	
Education level			0.10
None	44.1	111	
Primary	55.6	378	
Secondary or higher	51.9	160	
Work in the last 12 months			**< 0.001**
No	45.8	308	
Yes	58.9	341	
Religion			0.09
Catholic	49.8	279	
Anglican	50	208	
Muslim	62.6	99	
Others	58.7	63	
Currently in union			**< 0.01**
No	45.8	262	
Yes	57.4	387	
Number of wives or husbands			**< 0.01**
One	54.9	304	
Two plus	66.3	83	
Not in union	45.8	262	
Correct Knowledge about 4–5 HIV transmission modes			**0.01**
No	46.1	230	
Yes	56.3	419	
Stigma on at least 4–8 statements			0.71
No	53.2	436	
Yes	51.6	213	
Test for HIV once a year even HIV negative			**0.01**
No	31.4	35	
Yes	53.9	614	
Total	**52.7**	**100**	
Older men need to circumcise to prevent HIV			**0.01**
No	44.6	202	
Yes	56.4	447	
Had sex in the last 12 months			**< 0.001**
No	38.9	314	
Yes	66.9	320	
Number of lifetime sexual partners			0.39
One	65.4	231	
Two or more	70.5	88	
Ever given or received money or gifts for sex			0.06
No	50.6	490	
Yes	59.1	159	
Gave or received money or gifts for sex in the last 12 months			**0.01**
No	50.9	574	
Yes	66.7	75	
Drinks alcohol			0.94
No	52.6	426	
Yes	52.9	223	
Uses tobacco or drugs			0.33
No	52	564	
Yes	57.6	85	
Self-reported STI in the last 12 months			**< 0.01**
No	48.8	459	
Yes	62.1	190	
Male circumcised			**< 0.01**
No	48.2	199	
Yes	67.2	116	
No, female	50.3	334	
Total	**52.7**	**100**	

HIV testing prevalence declined with increase in age (64% for 50–59 compared to 19% of 80+ year olds; *p* < 0.001). Higher proportion of HIV testing were observed among respondents that worked in the past year (59%; *p* < 0.001), currently in union (57%; *p* < 0.01), polygamous unions (66%; *p* < 0.01), had correct knowledge of HIV transmission on 4–5 aspects (56%; *p* = 0.01), felt the need to test for HIV annually (54%; p = 0.01), felt need for male circumcision (56%; p = 0.01), were actually circumcised (67%; p < 0.01), had sex in the last 12 months (67%; p < 0.001), had transactional sex (67%; p = 0.01) in the last 12 months, and self-reported STIs in the past year (62%; p < 0.01). Education, religion, stigma, number of lifetime sexual partners, alcohol and drug use were not significantly associated with HIV testing 12 months preceding the study.

### Multivariable results

Table 3 shows the association between recent HIV testing and background factors among older persons in rural Uganda.

**Table 3:** Logistic regression of recent HIV testing against background factors, HIV knowledge and stigma and behavioural factors

Variables	Model 1		Model 2		Model 3	
	Adjusted Odds Ratios (aOR)	95% Confidence Interval	Odds Ratios (aOR)	95% Confidence Interval	Odds Ratios (aOR)	95% Confidence Interval
Age group						
50–59 (rc)	1				1.00	
60–69	0.61^**^	0.41–0.88	0.62^*^	0.43–0.92	0.78	0.52–1.18
70+	0.32^***^	0.22–0.49	0.36^***^	0.24–0.56	0.49^**^	0.31–0.79
Sex (rc = female)	1.08	0.75–1.55	1.11	0.77–1.59	0.79	0.47–1.13
Worked in the last 12 months (rc = No)	1.38^*^	1.00–1.93	1.38	1.01–2.10	1.34	0.96–1.91
Currently in union (rc = not in union)	1.46^*^	1.0102.09	1.46^*^	0.93–1.98	0.81	0.51–1.28
Correct knowledge on 4–5 HIV prevention modes			1.25	0.87–1.78	1.10	0.75–1.59
Agreement with on 4–8 stigma statements			1.03	0.72–1.47	1.01	0.69–1.45
Older men need to circumcise to prevent HIV (rc = No)			1.36	0.95–1.94	1.19	0.81–1.75
Self-reported sexually transmitted infections in the last 12 months (rc = No)					1.59^*^	1.09–2.30
Male circumcised						
No (rc)					1.00	
Yes					1.71^*^	1.00–2.93
Not Applicable to females					1.00	
Sexual activity in the last 12 months (rc = No)					2.89^***^	1.83–4.57
Transactional sex in the last 12 months (rc = No)					1.05	0.60–1.84
*Observations (N)*	646		646		631	

*aOR (Adjusted Odds Ratios); rc: reference category; 95% confidence intervals in second column:* ^*^ *p < 0.05,* ^**^ *p < 0.01,* ^***^ *p < 0.001;*

*Model 1, controlling for socio-demographics, Model 2, controlling for HIV knowledge and stigma & Model 3, controlling for behavioural factors*

Age was consistently associated with HIV testing in the past 12 months. Older persons age 70 and older consistently had reduced odds of testing for HIV compared to 50–59 year olds after adjusting for background factors (aOR = 0.33; 95% CI: 0.22–0.50), adding knowledge factors and attitudes (aOR = 0.37; 95% CI: 0.24–0.56) and finally adding behavioural factors (aOR = 0.49; 95% CI: 0.31–0.79).

In addition, being circumcised, sexual activity, transactional sex, and self-reported STIs in the past year, being circumcised were significantly associated with recent HIV testing. The odds of HIV testing increased (aOR = 1.59; 95% CI: 1.0–2.3) among respondents who had a self-reported STI compared to those that did not. Older men who were circumcised compared to those (older men) who were not were more likely (aOR = 1.71; 95% CI: 1.0–2.9) to test for HIV in the last 12 months. Also, those who had sex in past year compared to those that did not, had increased odds (aOR = 2.89; 95% CI: 1.8–4.6) of HIV testing.

Working in the past year was significantly associated (aOR = 1.39; 95% CI: 1.0–1.9) with HIV testing after adjusting for background characteristics but subsequently lost its influence after adjusting for the rest of the explanatory factors (Models 2 and 3). Likewise, the odds of HIV testing among 60–69 year olds reduced in the first and second models compared to 50–59 year olds but were not significant in the third model. The association between sex, number of spouses, knowledge of HIV transmission modes, stigma, acknowledging the need for circumcision among older person and engaging in transactional sex 12 months prior to the study were not significantly associated with HIV testing 12 months prior to the study.

## Discussion

We set out to establish the prevalence and examine the determinants of HIV testing in the last 12 months among older persons in rural Uganda. Over half of (53%) older persons had tested HIV during the 12 months preceding the study. This finding is in consistent with the proportion (48%) of older persons (age 45–59 years) that had tested for HIV [37] in the 2011 Uganda AIDS indicator survey [26].

The determinants of HIV testing among old persons in the year preceding the study in order of strength of influence were: sexual activity in the past year, male circumcision, having a self-reported STIs and advanced age. Sexual activity in the last 12 months increased the odds of recent HIV testing. Among older persons, recent sexual activity increases perceived risk of HIV infection which motivates older persons to have an HIV test [38, 45]. This is because sexual activity is one of the main avenues of HIV transmission [57]. Older men tend to remain sexually active and engage in extra-marital affairs more than older women [35].

Our study found that self-reported STIs were positively associated with HIV testing. Self-reported STIS are indicative of engagement in risky sexual behaviours that increase the odds of HIV infection. HIV testing in such cases is through risk perception or referral by health providers [45].

The study found that circumcised older persons had increased odds of HIV testing [58]. The possible explanation is that in case of medical circumcision, it is possible that older persons who interacted with the health sector also benefited from HIV relevant health education [59]. In addition, they might choose not to use condoms and instead prefer to test for HIV.

This study found that advanced age reduced odds of testing for HIV. This finding is in agreement with studies elsewhere [44]. This could be associated with low perceived HIV risk, and lack of associated information [38, 45].

This study merits the following strengths: first, it highlights important findings about HIV testing among older persons in Uganda using quantitative survey data with a good sample size. The findings provide a benchmark for conducting further studies in Uganda.

None the less, there are some limitations of the data. First, it is cross-sectional data and we cannot easily ascertain the direction of causality of associations between HIV testing and self-reported STIs and sexual activity in the last 12 months. Finally, HIV knowledge reliability score of 0.43 showed that the statements are not very reliable.

## Conclusion

Recent HIV testing among older persons is associated with younger age (50–59 years), self-reported STIs, male circumcision, and sexual activity among older persons in rural Uganda. HIV testing interventions need to target older persons age 70 years and older, who were less likely to test. These interventions include behavioral risk assessment and routine screening for HIV infection.

## Data Availability

The datasets generated and analysed during the current study are not publicly available due for confidentiality reasons but are available from the corresponding author on reasonable request.
